# Increasing the intracellular availability of all-*trans* retinoic acid in neuroblastoma cells

**DOI:** 10.1038/sj.bjc.6602398

**Published:** 2005-02-15

**Authors:** J L Armstrong, M Ruiz, A V Boddy, C P F Redfern, A D J Pearson, G J Veal

**Affiliations:** 1 Northern Institute for Cancer Research, Paul O'Gorman Building, Medical School, Framlington Place, University of Newcastle Upon Tyne, Newcastle Upon Tyne NE2 4HH, UK

**Keywords:** retinoic acid, CYP26, R116010, cellular retinoic acid binding protein, acitretin

## Abstract

Recent data indicate that isomerisation to all-*trans* retinoic acid (ATRA) is the key mechanism underlying the favourable clinical properties of 13-*cis* retinoic acid (13cisRA) in the treatment of neuroblastoma. Retinoic acid (RA) metabolism is thought to contribute to resistance, and strategies to modulate this may increase the clinical efficacy of 13cisRA. The aim of this study was to test the hypothesis that retinoids, such as acitretin, which bind preferentially to cellular retinoic acid binding proteins (CRABPs), or specific inhibitors of the RA hydroxylase CYP26, such as R116010, can increase the intracellular availability of ATRA. Incubation of SH-SY5Y cells with acitretin (50 *μ*M) or R116010 (1 or 10 *μ*M) in combination with either 10 *μ*M ATRA or 13cisRA induced a selective increase in intracellular levels of ATRA, while 13cisRA levels were unaffected. CRABP was induced in SH-SY5Y cells in response to RA. In contrast, acitretin had no significant effect on intracellular retinoid concentrations in those neuroblastoma cell lines that showed little or no induction of CRABP after RA treatment. Both ATRA and 13cisRA dramatically induced the expression of CYP26A1 in SH-SY5Y cells, and treatment with R116010, but not acitretin, potentiated the RA-induced expression of a reporter gene and CYP26A1. The response of neuroblastoma cells to R116010 was consistent with inhibition of CYP26, indicating that inhibition of RA metabolism may further optimise retinoid treatment in neuroblastoma.

Retinoic acid (RA) plays an important role in the growth and differentiation of a variety of cell types, and can reverse malignant growth *in vitro* and *in vivo*. Retinoic acid or synthetic analogues have been used with some success as chemotherapeutic agents, particularly in the treatment of neuroblastoma (NBL) (Reynolds *et al*, 2003).

Neuroblastoma is the most common extracranial malignant solid tumour of childhood, and cure rates, especially for stages 3 and 4, remain low. Studies comparing the activity of 13-*cis* retinoic acid (13cisRA) and all-*trans* retinoic acid (ATRA) in NBL cell lines have demonstrated similar potency of these retinoids, in terms of cellular differentiation, growth arrest and regulation of markers such as MYCN (Thiele *et al*, 1985; Reynolds *et al*, 1994; Veal *et al*, 2002). The unfavourable pharmacokinetic properties of ATRA have limited its use in the treatment of NBL (Reynolds *et al*, 2003). In contrast, peak drug levels achieved in NBL patients receiving 13cisRA given as high-dose pulse therapy have been shown to be above the 5 *μ*M concentration required to inhibit the growth of NBL cells *in vitro* (Villablanca *et al*, 1995). A further trial showed that 13cisRA improved event-free survival in advanced stage NBL patients when given after either autologous bone marrow transplantation (ABMT) or nonmyeloablative therapy, that is, in a setting of minimal residual disease; however, approximately 50% of patients develop resistance or are unresponsive to 13cisRA therapy (Matthay *et al*, 1999).

Several lines of evidence suggest that 13cisRA acts as a prodrug for ATRA. 13cisRA has a much lower binding affinity than ATRA for the retinoic acid receptor (RAR) family of ligand-activated transcription factors (Åstrom *et al*, 1990; Allenby *et al*, 1993; Idres *et al*, 2002). Furthermore, intracellular accumulation of ATRA after incubation with 13cisRA and a delayed response to 13cisRA compared to ATRA is seen in both NBL and sebocyte cell lines, and in a human NBL xenograft model, supporting this hypothesis (Tsukada *et al*, 2000; Ponthan *et al*, 2001; Veal *et al*, 2002). Little is known about the mechanisms of RA isomerisation *in vivo*, although a role for thiol groups has been suggested (Lanvers *et al*, 1998).

Metabolism may also be important in maintaining effective ATRA levels. Retinoic acid can be oxidised by cytochrome *P*450 enzymes to less active metabolites including 4-oxo-RA, 4-OH-RA, 18-OH-RA and 5,6-epoxy-RA (Marill *et al*, 2000, 2002). Novel RA-inducible *P*450s, termed *P*450RAIs or CYP26s, have recently been characterized, which have high specificity for ATRA (White *et al*, 1996, 2000; Tahayato *et al*, 2003). Cells transfected with full-length CYP26 accumulate polar metabolites, suggesting that CYP26 isoforms play an important role in ATRA metabolism. Moreover, a clear relationship between induction of RA metabolism and progressive clinical resistance to RA has been demonstrated in acute promyelocytic leukaemia patients (Muindi *et al*, 1992). A novel inhibitor of RA metabolism, R116010, has recently been shown to inhibit the oxidation of ATRA, enhance the biological activity of ATRA *in vitro* and exhibit antitumour activity *in vivo* (Van heusden *et al*, 2002).

The metabolism of ATRA is also thought to be regulated by cellular retinoic acid binding proteins (CRABPs), which may be involved in the cellular transport of ATRA (Dong *et al*, 1999). Induction of CRABPs has been reported in a number of cell lines in response to RA (Redfern *et al*, 1994; Plum and Clagett-Dame, 1995). Cellular retinoic acid binding protein I has a higher affinity for ATRA than that of CRABP II and the rate of ATRA metabolism in isolated microsomes is faster in the presence of CRABP I than without, suggesting a direct role for CRABP I in ATRA metabolism (Fiorella and Napoli, 1991; Fogh *et al*, 1993). Furthermore, the rate of formation of polar metabolites of RA in intact cells is determined by the level of CRABP I (Boylan and Gudas, 1992). On this basis, cellular retinoic acid binding protein I appears to facilitate retinoid degradation. Acitretin is an analogue of RA used clinically to treat psoriasis and malignant skin disorders (McNamara *et al*, 2002; Geiger, 2003); it has a high affinity for both CRABP I and II, but unlike ATRA, acitretin has a much lower affinity for the RARs. Acitretin is known to increase the occupancy of the RAR-transcription complex with ATRA by displacement of ATRA from CRABPs (Tian *et al*, 1997), and it has been proposed that the responsiveness of tissue culture cell lines to acitretin may correlate with CRABP expression.

Although the catabolism of ATRA and 13cisRA potentially limits clinical use, agents that inhibit ATRA metabolism, such as acitretin or CYP26 inhibitors, may increase its biological availability and directly affect the efficacy of retinoid treatment. Therefore, to facilitate the continued development of RA for NBL therapy, the aim of this study was to test the hypothesis that acitretin and R116010 increase the retinoid response of NBL cells to treatment with ATRA or 13cisRA.

## MATERIALS AND METHODS

### Cell lines

SH-SY5Y, SH-EP (N- and S-type cells, respectively, derived from the parental line SK N SH), IMR-32 and NGP NBL cell lines were cultured routinely in RPMI 1640 medium containing foetal calf serum (10%), L-glutamine (2 mM), penicillin (100 U ml^−1^) and streptomycin (100 *μ*g ml^−1^). Cells were grown at 37°C in a humidified incubator containing 5% CO_2_ and routinely tested negative for mycoplasma.

### Chemicals

Retinoic acid isomers were purchased from Sigma (Poole, UK). Acitretin (Ro 10-1670) was generously provided by Hoffmann-La Roche, Basal, Switzerland. HPLC grade *n*-hexane, glacial acetic acid and propan-2-ol were from Fisher Scientific (Loughborough, UK). Protease inhibitor cocktail was from Roche (Lewes, UK). R116010 was a gift from Barrier Therapeutics Inc. (Princeton, NJ, USA).

### Retinoid treatment

Retinoids were dissolved in dimethyl sulphoxide (DMSO) and diluted in cell culture medium to obtain final concentrations of 10^−10^–10^−4^ M. The final concentration of DMSO in all experiments never exceeded 0.2%. All experiments were performed in dim light and tubes containing retinoids were wrapped in aluminium foil.

### Extraction of retinoids

Cell pellets were resuspended in 2 ml cell culture medium and disrupted by passage several times through a hypodermic needle (25G × 1 in, 0.5 mm × 25 mm; Terumo, Somerset, NJ, USA). Proteins were precipitated by adding 500 *μ*l ethanol and 500 *μ*l saturated ammonium sulphate solution to 2 ml of extracellular medium or resuspended cells. Samples were vortex mixed and retinoids extracted into *n*-hexane, dichloromethane and propan-2-ol (80 : 19 : 1) on a rotary mixer for 8 min. After centrifugation at 2000 **g** for 30 min at 4°C, the organic layer was removed, evaporated under a stream of nitrogen and analysed by HPLC (detection limits 0.02–4 *μ*g ml^−1^) (Veal *et al*, 2002).

### HPLC analysis

Samples were reconstituted in 200 *μ*l mobile phase A and intra- and extracellular retinoid levels were quantified by HPLC analysis using a Waters 2690 Separations Module and 996 Photodiode array (PDA) detector (Waters Ltd., Elstree, UK), with Waters Millennium software for data acquisition, as described previously (Veal *et al*, 2002). Cell volumes were determined as described previously (Lu *et al*, 2000) and RA concentrations converted from *μ*g ml^−1^ lysate to *μ*M intracellular concentrations (Veal *et al*, 2002).

### Preparation of cell extracts for western blotting

Cells were washed three times with ice-cold phosphate-buffered saline and scraped into extraction buffer (100 mM Tris-HCl, pH 7.4, 100 mM KCl, 2 mM EDTA, 25 mM KF, 0.1% (v/v) Triton X-100, 1 mM benzamidine, 0.1 mM Na_3_VO_4_, 1 *μ*g ml^−1^ pepstatin, and complete protease inhibitor cocktail), transferred to 1.5 ml Eppendorf tubes, and immediately frozen in liquid nitrogen. Prior to analysis, samples were thawed and dispersed by sonication for 1 min, and the protein concentration determined by a dye-binding method (Bradford, 1976).

### Western blot analysis

Whole-cell extracts (10 *μ*g of protein) were loaded onto polyacrylamide gels, electrophoresed, and blotted onto polyvinylidene difluoride membranes (PVDF, Bio-Rad, Hemel Hempstead, UK). Membranes were blocked with 5% nonfat milk in Tris-buffered saline (TBS), pH 7.6, 0.1% Tween 20 (TBS-T), then incubated with antibodies to CRABP I (S-14) or CRABP II (K-13) (Santa Cruz Biotechnology, Santa Cruz, CA, USA) or *β*-actin (Sigma, Poole, UK). The antibody to CRABP I was generated from a peptide corresponding to an internal region of human CRABP I. Membranes were incubated with biotin-labelled anti-goat IgG followed by streptavidin-linked horseradish peroxidase (HRP) (Amersham Biosciences, Chalfont St Giles, UK) at 1 : 1000 dilution, or HRP-coupled anti-mouse IgG (Upstate Ltd, Milton Keynes, UK) at 1 : 15 000 dilution for 1 h at room temperature. Each step was followed by at least four 10-min washes in TBS-T. ECL-plus detection was carried out according to the manufacturer's instructions (Amersham Biosciences) and band intensity analysed by phosphorimager (Storm 860).

### Cell proliferation studies

Cells were seeded in 96-well plates at a density of 2500 cells well^−1^ and allowed to attach overnight. 13-*cis* retinoic acid, ATRA, acitretin or R116010 were added to a final concentration of 0.01–100 *μ*M. Growth was determined after 6 days using the SRB assay as described previously (Skehan *et al*, 1990). Briefly, cells were fixed with 10% trichloroacetic acid (TCA) and stained with 0.4% sulphorhodamine B (SRB) in 1% acetic acid for 30 min. Protein-bound dye was extracted with 10 mM Tris (unbuffered) and absorption measured at 570 nm.

### Transient cell transfections for transactivation studies

The day before transfection, cells were seeded into 24-well culture plates at a density of 6.5 × 10^4^ cells per well. Cells were cotransfected, using FuGENE 6 reagent (Roche, Lewes, UK) according to the manufacturer's instructions, with 0.4 *μ*g of reporter vector pTA-RARE-SEAP or the pTA-SEAP negative control vector (BD Biosciences Clontech, Oxford, UK), and 0.1 *μ*g pGL3 vector that encodes the *Photinus pyralis* luciferase (Promega, Southampton, UK) as an internal control to normalise for variations in transfection efficiency. At 24 h after transfection, cells were treated with the appropriate retinoid at the indicated concentrations and times. SEAP (Applied Biosystems, Warrington, UK) and luciferase (Promega) detection were carried out according to the manufacturer's instructions.

### Reverse-transcriptase polymerase chain reaction

Total RNA was isolated using the RNeasy Mini Kit including DNase digestion (Qiagen, Crawley, UK). Reverse transcriptase polymerase chain reaction (RT–PCR) was performed using the Access RT–PCR kit (Promega) with primers for CYP26A1 (Kim *et al*, 2002) or *β*-actin (sense 5′-GGCATCCTCACCCTGAAGTA-3′; antisense 5′-GATCTGGGTCATCTTCTCGC-3′). In all, 0.5 *μ*g (for CYP26A1 amplification) or 0.005 *μ*g (for *β*-actin amplification) RNA was used in each reaction and 40 cycles of PCR were performed under the following conditions: 94°C, 30 sec; 65°C, 1 min; 72°C, 2 min; and a final extension step at 72°C, 7 min. Under these conditions, the PCR reactions were within the linear range for amplification of CYP26A1 and *β*-actin, respectively. Control reactions (no RNA, no reverse transcriptase) were performed according to the manufacturer's instructions, and the CYP26A1 PCR product was sequenced to confirm identity. PCR products were separated on a 2% (w/v) agarose gel, and visualized by UV light illumination after ethidium bromide staining.

### Real-time PCR – CYP26A1

RNA was reverse-transcribed using Promega's Reverse Transcription System according to the manufacturer's instructions, using random hexamer primers. Real-time PCR was performed on 20 ng cDNA using TaqMan Assays-on-Demand Gene Expression products for human CYP26A1 in combination with the TaqMan Universal PCR master mix (Applied Biosystems). Appropriate controls for nonspecific amplification and contamination were included. A GeneAmp 5700 Sequence Detection System was used for real-time PCR amplification. As internal standard, *β*-actin was measured simultaneously using the endogenous control assay provided by Applied Biosystems. PCR amplification procedures followed manufacturer's instructions. Briefly, the thermocycling program consisted of one cycle at 50°C for 2 min followed by 95°C for 10 min and 40 cycles at 95°C (15 s) and 60°C (1 min). The data were analysed using the GeneAmp Sequence Detection System software.

### Statistical analysis

Analysis was performed using ANOVA or *t*-tests. For experiments involving measurements of retinoid concentrations or SEAP activation, the variance increased in proportion to the mean and statistical analysis was therefore performed after log transformation of the data. For specific comparisons (hypothesis tests) between treatments, Bonferroni correction (Bland, 2000) was used to maintain an experiment-wise probability for significance of 0.05. Linear contrasts were used to test for dose–response relationships within the ANOVA models.

## RESULTS

### Effect of acitretin on intracellular retinoid concentrations

**Figure 1:**
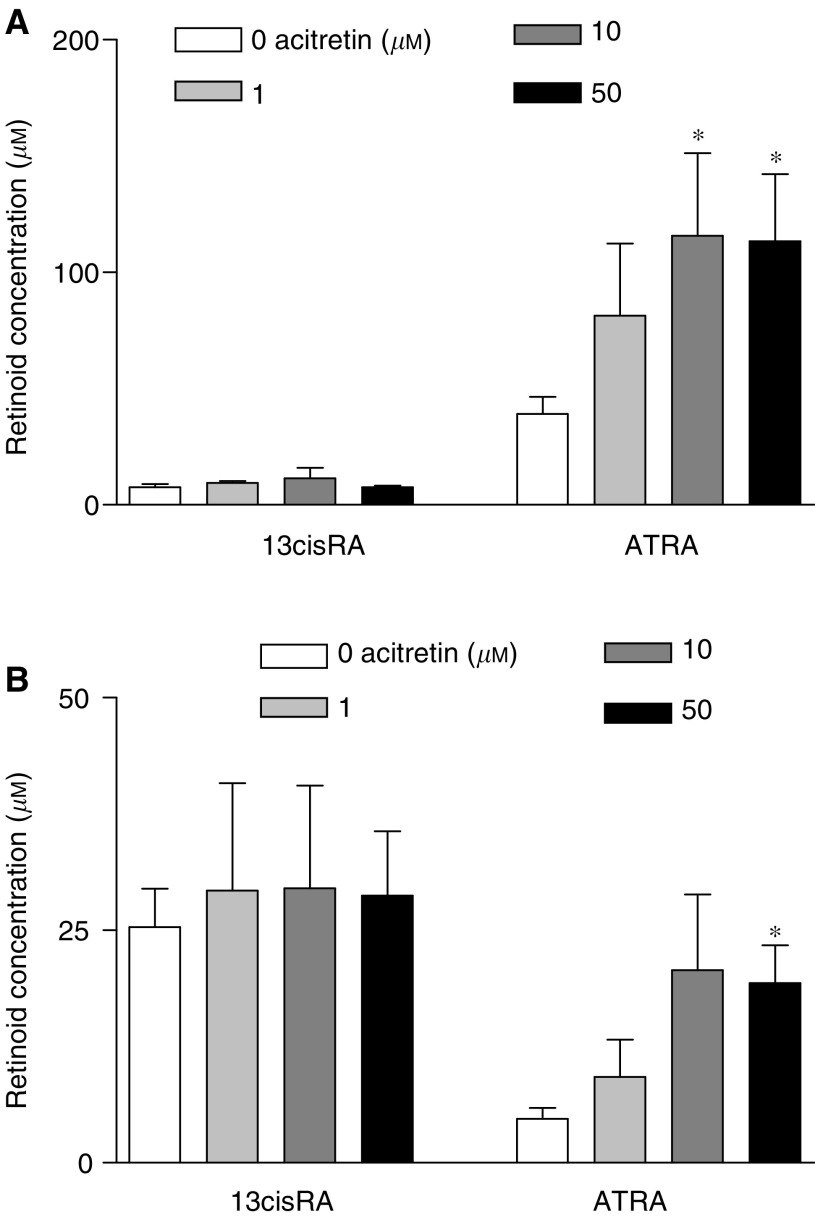
SH-SY5Y cells were incubated with acitretin (0–50 *μ*M) for 24 h prior to the addition of 10 *μ*M ATRA (**A**) or 10 *μ*M 13cisRA (**B**) for a further 24 h. Values are expressed as intracellular retinoic acid (RA) concentrations based on cell counts and cell volume calculations. Data are mean values±s.e.m. from at least three separate experiments. Statistical significance relative to controls without acitretin is indicated by ^*^(*P*<0.05). For the ANOVA with intracellular ATRA levels in (**B**) (F_3,13_=5.078, *P*=0.015), one outlier for the 10 *μ*M acitretin dose was removed (studentised residual −2.682).

SH-SY5Y, SH-EP and IMR-32 NBL cells were treated with ATRA or 13cisRA (10 *μ*M) for 24 h. The RA isomer present initially was the predominant isomer observed following 24 h incubation, as demonstrated in SH-SY5Y cells (Figure 1). Concentrations of total intracellular retinoids were lower after incubation with 13cisRA than after incubation with ATRA, suggesting isomer-specific uptake.

Preincubation for 24 h with a range of acitretin concentrations prior to RA incubation for a further 24 h, significantly increased intracellular ATRA concentrations in SH-SY5Y cells incubated with ATRA (one-way ANOVA, F_3,15_=3.9, *P*=0.03): acitretin concentrations of 10 and 50 *μ*M significantly increased intracellular ATRA levels relative to controls without acitretin, from 39.1±7.4 (mean±s.e.m.) *μ*M with ATRA alone to 115.7±35.5 *μ*M in the presence of 10 *μ*M acitretin and 113.5±28.7 *μ*M with 50 *μ*M acitretin, respectively (Figure 1A) (F_1,15_⩾7.2, *P*⩽0.017, significant at *P*⩽0.05 after Bonferroni correction). For cells incubated with 13cisRA, 50 *μ*M acitretin significantly increased intracellular ATRA levels relative to controls without acitretin, from 4.2±1.1 *μ*M with 13cisRA alone to 19.3±4.1 *μ*M (Figure 1B) (F_1,13_=10.503, *P*=0.006). Conversely there was no effect of acitretin in increasing intracellular levels of 13cisRA in cells incubated with ATRA (one-way ANOVA F_3,15_=0.315, *P*>0.8) or 13cisRA (F_3,12_=0.047, *P*>0.9). Extracellular concentrations of ATRA and 13cisRA in the medium from cells pretreated with or without 1–100 *μ*M acitretin were not significantly different (ATRA incubations: 13cisRA levels F_3,8_=0.9, *P*=0.46; ATRA levels F_3,8_=1.35, *P*=0.33 and 13cisRA incubations: 13cisRA levels F_3,8_=3.38, *P*=0.08; ATRA levels F_3,8_=0.44, *P*=0.73).

The effect of 50 *μ*M acitretin was also assessed in SH-SY5Y, SH-EP, IMR-32 and NGP cell lines over a time course of 6–72 h (data not shown). In each cell line, peak intracellular ATRA concentrations in the presence of acitretin were observed after 24 h (Table 1 ). However, in contrast to SH-SY5Y cells, where a selective increase in intracellular ATRA concentrations was observed, pretreatment of SH-EP, IMR-32 or NGP cells with acitretin, prior to incubation with ATRA or 13cisRA, did not result in a significant increase in either ATRA or 13cisRA intracellular levels (Table 1).

### Analysis of CRABP expression

**Figure 2:**
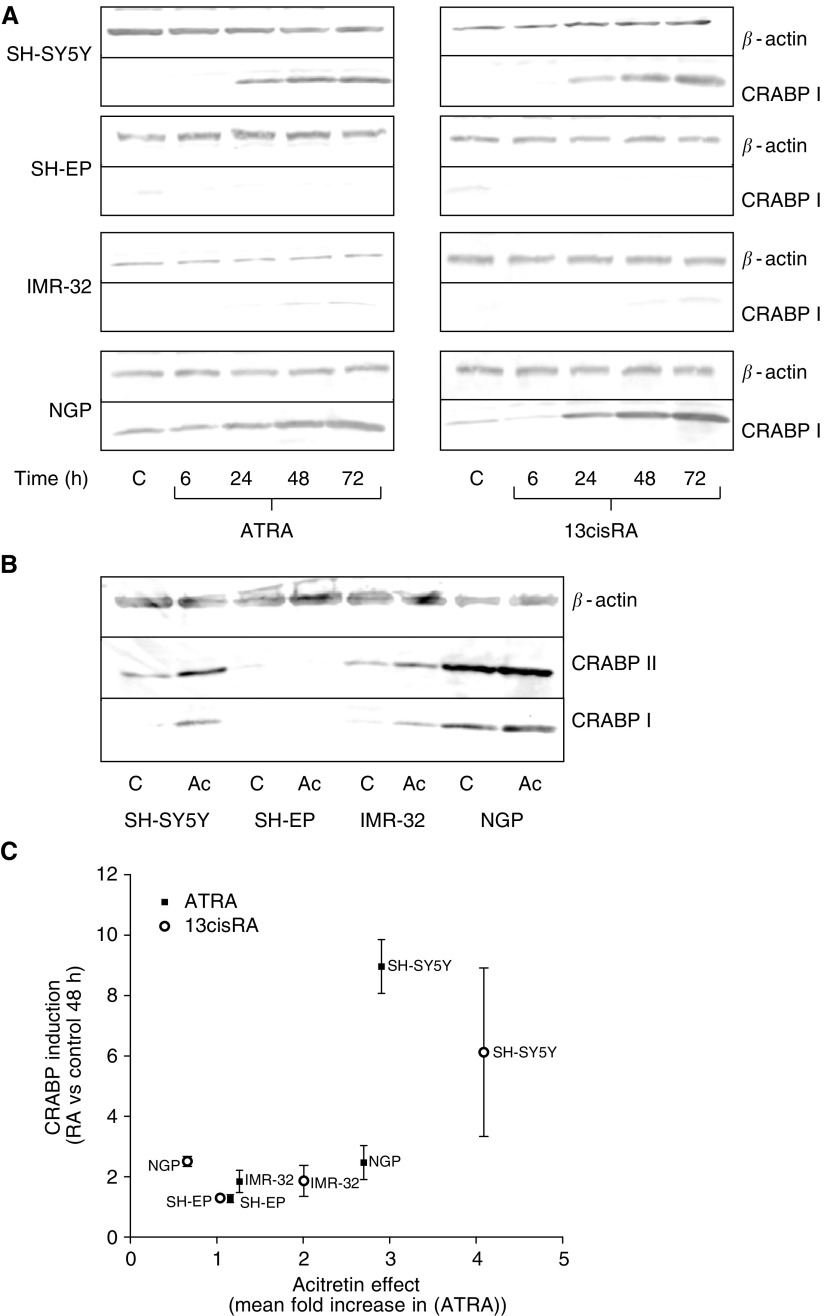
CRABP I/II expression determined by Western blot analysis in SH-SY5Y, SH-EP and IMR-32 cells after 0–72 h incubations with ATRA or 13cisRA (10 *μ*M) (**A**) and after 48 h incubation with acitretin (Ac, 50 *μ*M) (**B**). *β*-Actin was used as a reference to correct for protein loading. (**C**) Graph plotting CRABP I induction after 48 h incubation with ATRA or 13cisRA (10 *μ*M) against the mean increase in intracellular ATRA concentration for each cell line from Table 1 (control compared to acitretin treated cells).

It was hypothesised that the responsiveness of tissue culture cell lines to acitretin may correlate with CRABP expression and therefore the relationship between CRABP expression and the effect of acitretin on intracellular ATRA levels was evaluated. Proteins detected by the CRABP I antibody (here referred to as CRABP I) in SH-SY5Y, SH-EP, IMR-32 and NGP cells treated with ATRA or 13cisRA (10 *μ*M) for 0–72 h were evaluated by Western blotting (Figure 2A). CRABP I expression was induced by ATRA and 13cisRA in a time-dependent manner in SH-SY5Y, and minimally in IMR-32 cells. Cellular retinoic acid binding protein I expression was induced in NGP cells after treatment with either ATRA or 13cisRA, although expression was also detected in untreated cells. CRABP I was not detected in SH-EP cells after incubation with either ATRA or 13cisRA. There was induction of CRABP I expression in SH-SY5Y and IMR-32 cells treated with 50 *μ*M acitretin for 48 h, but little induction in NGP cells and none in SH-EP cells. Similar results were obtained using an antibody to CRABP II (Figure 2B). These results suggest that the effect of acitretin on intracellular ATRA levels reflected the induction of expression of CRABP proteins in these cell lines, indicated by a graph plotting CRABP I induction after 48 h incubation with ATRA or 13cisRA (10 *μ*M) against the mean increase in intracellular ATRA concentration for each cell line from Table 1 (control compared to acitretin-treated cells) (Figure 2C).

### Effect of R116010 on intracellular retinoid concentrations

**Figure 3:**
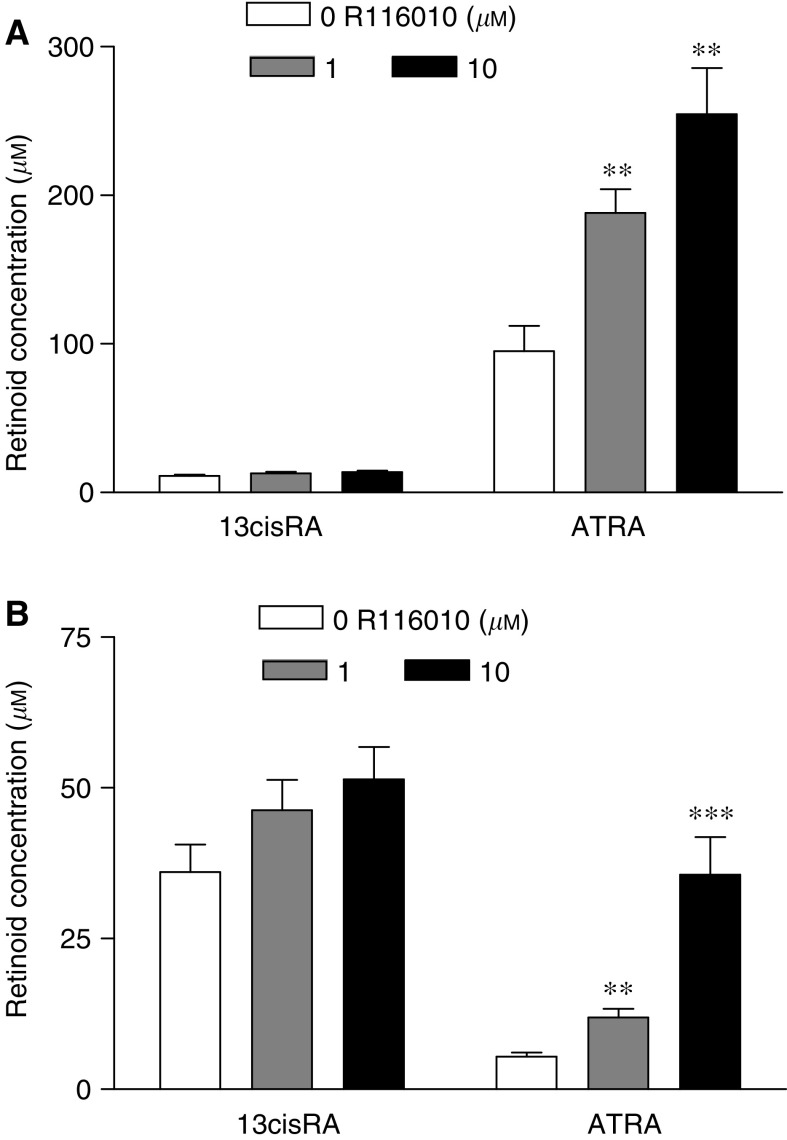
SH-SY5Y cells were coincubated with R116010 (0–10 *μ*M) and either ATRA (**A**) or 13cisRA (**B**) (10 *μ*M) for 24 h. Values are expressed as intracellular retinoic acid (RA) concentrations based on cell counts and cell volume calculations. Data plotted are mean values±s.e.m. from at least four separate experiments. Statistical significance relative to controls without R116010 is indicated by ^***^ for *P*<0.001 and ^**^ for *P*<0.01.

The effect of R116010 was examined in SH-SY5Y cells, a cell line known to metabolise ATRA rapidly over 24 h. Coincubation of R116010 and either ATRA or 13cisRA in SH-SY5Y cells for 24 h significantly increased intracellular ATRA levels (one-way ANOVA, F_2,15_⩾11.5, *P*⩽0.001), and this increase was approximately linear with respect to R116010 dose (Linear contrasts, F_1,15_>12, *P*⩽0.003; Figure 3A and B). Treatment with R116010 resulted in 2- and 2.7-fold increases in ATRA concentrations from 95.2±16.9 (mean±s.e.m.) *μ*M with ATRA alone to 188±19 *μ*M in the presence of 1 *μ*M R116010 and 254.7±31 *μ*M with 10 *μ*M R116010, respectively (Figure 3A). After incubation with 13cisRA, R116010 gave 2.2- and 6.5-fold increases in intracellular ATRA concentrations from 5.4±0.7 *μ*M with 13cisRA alone to 11.9±1.4 and 35.6±6.2 *μ*M with 1 and 10 *μ*M R116010, respectively (Figure 3B). Conversely, there was no effect of R116010 on intracellular 13cisRA concentrations in cells incubated in ATRA or 13cisRA (F_2,15_⩽2.213, *P*⩾0.144). Incubation with R116010 alone (1 and 10 *μ*M) did not result in concentrations of endogenous intracellular retinoids above the lower limit of detection of the HPLC assay (0.02–4 *μ*g ml^−1^).

### Effect of acitretin and R116010 on cell proliferation

Retinoic acid has previously been shown to inhibit the growth of NBL cells in culture. To determine whether the use of inhibitors of RA metabolism could amplify this effect, the SRB assay was used to assess the effect of acitretin and R116010 on SH-SY5Y cell proliferation (Table 2 ). R116010 (1 *μ*M) had little effect on cell growth, whereas acitretin (1 *μ*M) had a substantial effect, inhibiting proliferation to 66.5 of control cell growth. The effect of acitretin alone therefore masked any potentiation of RA-induced growth inhibition. In contrast R116010 (1 *μ*M) potentiated the growth inhibitory effects of ATRA and 13cisRA at concentrations of 0.01 and 0.1 *μ*M, respectively (one-way ANOVA hypothesis tests, F_1,27_=8.248, *P*=0.008 for ATRA and F_1,27_=17.579, *P*<0.001 for 13cisRA). However, the overall degree of potentiation by R116010 was relatively modest (from 60.3 to 48.9% for ATRA and from 53.8 to 42% for 13cisRA).

### Effect of acitretin and R116010 on markers of retinoid response

**Figure 4:**
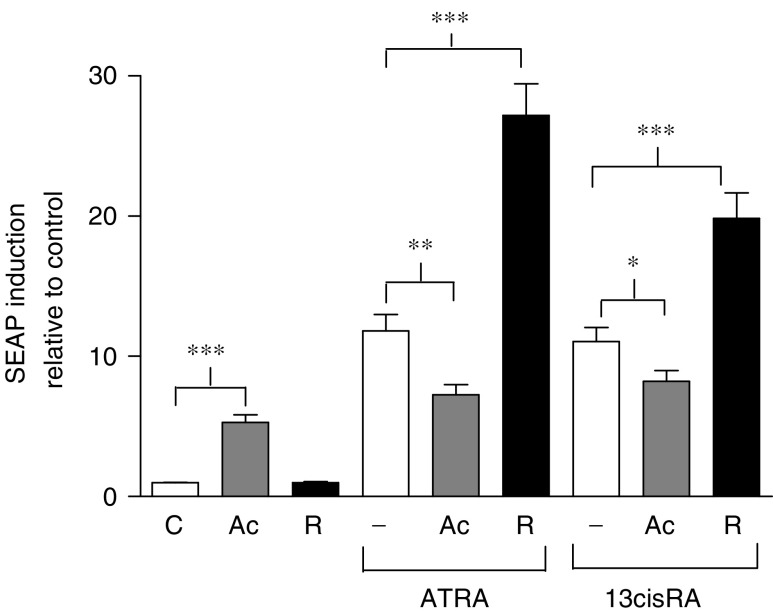
Induction of SEAP expression. SH-SY5Y cells were incubated with ATRA or 13cisRA (0.1 *μ*M) for 48 h in the absence or presence of acitretin (Ac, 1 *μ*M) or R116010 (R, 1 *μ*M). Values are expressed as fold increase relative to expression in untreated control cells. Data are mean values±s.e.m. from at least three independent experiments. Statistical significance from hypothesis tests in ANOVA is indicated by ^***^ for *P*<0.001, ^**^ for *P*<0.01 and ^*^ for *P*<0.05.

Retinoid response was evaluated in the SH-SY5Y cell line, previously shown to have good induction of the retinoid responsive genes, CRABP II and RAR*β* (Veal *et al*, 2002). To assess the transcriptional activity of increasing intracellular ATRA concentrations, SH-SY5Y cells were transfected with the reporter gene ‘secreted alkaline phosphatase’ (SEAP) under control of a DR5 retinoic acid response element. The effect of acitretin or R116010 (1 *μ*M) in combination with ATRA or 13cisRA (0.1 *μ*M) was studied after a 48 h incubation period and analysed by two-way ANOVA for retinoid effects and acitretin/R116010 effects (Figure 4). Incubation with acitretin alone resulted in a five-fold increase in SEAP expression (F_1,43_=166.9, *P*<0.001). Incubation with either ATRA or 13cisRA resulted in an 11-fold increase in SEAP expression relative to untreated cells, but there was no significant difference in response between ATRA and 13cisRA (F_1,43_=0.235, *P*=0.63). R116010 alone had no effect on SEAP expression. In the presence of acitretin, induction of SEAP expression in response to ATRA or 13cisRA was significantly reduced relative to the effects of these retinoids alone (F_1,43_=8.83, *P*=0.005 and F_1,43_=4.042, *P*=0.05 for ATRA and 13cisRA, respectively). This apparent antagonistic effect of acitretin was also observed on CRABP II expression when comparing the effect of ATRA alone and in combination with acitretin. Incubation with ATRA (1 *μ*M) for 24 h induced a 5.3-fold increase in CRABP II expression compared to untreated control cells, whereas ATRA in combination with acitretin (10 *μ*M) induced a 3.5-fold increase in expression, as determined by Western blot analysis (*t*_6_=2.78, *P*=0.032).

All-*trans* retinoic acid or 13cisRA in combination with R116010 resulted in a 2.3- and 1.8-fold increase, respectively, in SEAP expression relative to each RA alone (F_1,43_=40.892, *P*<0.001 and F_1,43_=19.656, *P*<0.001 for ATRA and 13cisRA, respectively). Although there was no difference between the SEAP induction seen in response to ATRA compared to 13cisRA alone, the effect of R116010 on SEAP induction in response to ATRA was greater than that in response to 13cisRA (F_1,43_=4.951, *P*=0.031).

**Figure 5:**
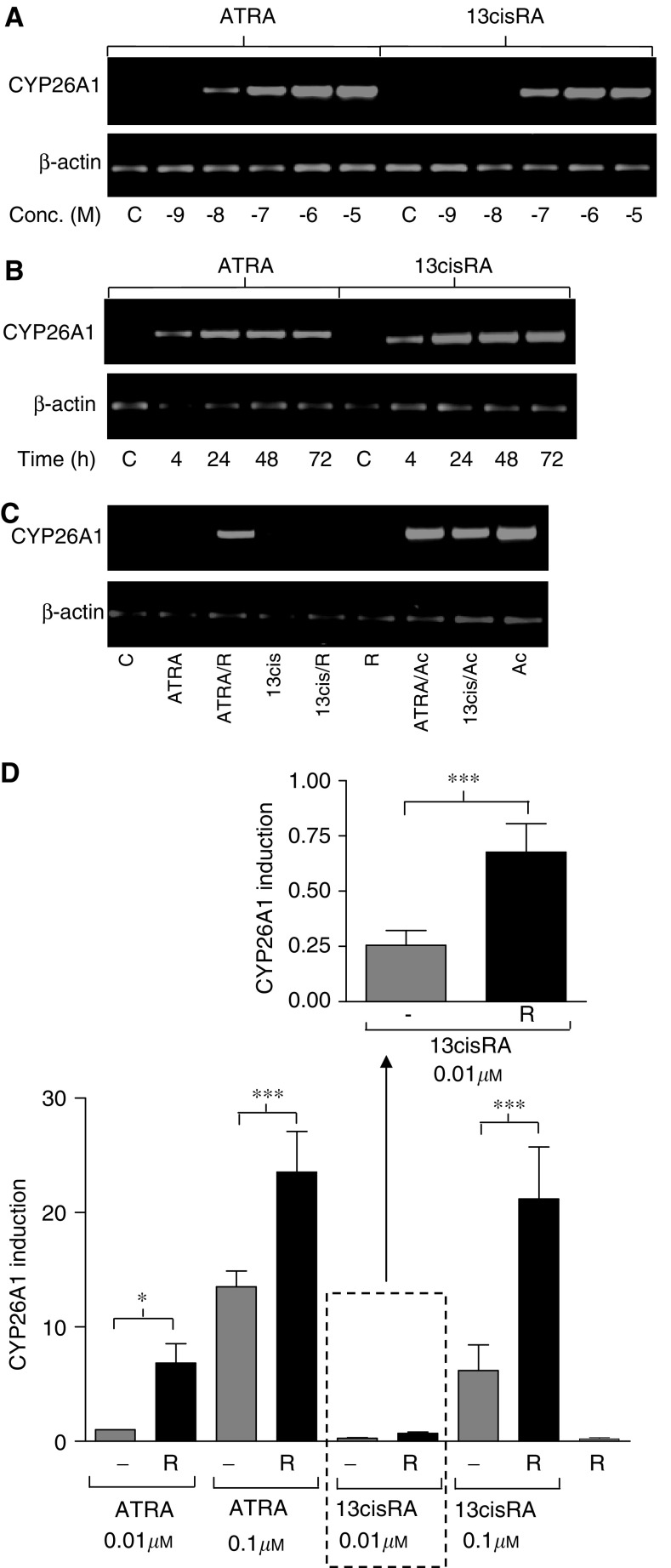
Induction of CYP26A1 mRNA expression determined by RT–PCR and real-time PCR. SH-SY5Y cells were treated with ATRA or 13cisRA for 24 h at the concentrations indicated (**A**) or with a fixed concentration of 1 *μ*M ATRA or 13cisRA for up to 72 h (**B**). SH-SY5Y cells were treated with ATRA or 13cisRA (0.01 *μ*M) for 24 h in the absence or presence of acitretin (Ac, 1 *μ*M) or R116010 (R, 1 *μ*M) (**C**). *β*-actin mRNA expression was used to normalise the reverse transcription reaction. Amplified products were analysed by agarose gel electrophoresis with ethidium bromide staining. Reverse-transcribed RNA from SH-SY5Y cells treated with ATRA or 13cisRA (0.01 or 0.1 *μ*M) for 24 h in the absence or presence R116010 (R, 1 *μ*M) was subjected to real-time PCR using TaqMan probes for CYP26A1 and *β*-actin (**D**). Values are normalised for *β*-actin levels and expressed as fold increase relative to expression of CYP26A1 in cells treated with 0.01 *μ*M ATRA. Data are mean values±s.e.m. from at least three independent experiments. Statistical significance from hypothesis tests in ANOVA is indicated by ^*^ where F_1,32_=5.714, *P*<0.05, and ^***^ where F_1,32_=15.496, *P*<0.001.

The expression of CYP26 may be an important marker of the response of NBL cells to retinoids, both *in vivo* and *in vitro*, and was measured using RT–PCR and real-time PCR. CYP26A1 expression was rapidly induced after incubation of cells with ATRA or 13cisRA in a time- and concentration-dependent manner (Figure 5A and B), with ATRA more potent than 13cisRA. Incubation with acitretin (1 *μ*M) resulted in a marked induction in CYP26A1 expression after 24 h, which was not increased further upon coincubation with either ATRA or 13cisRA (0.01 *μ*M, Figure 5C). Incubation with R116010 alone (1 *μ*M) had no effect on CYP26A1 expression, but R116010 (1 *μ*M) coincubated with either ATRA or 13cisRA (both at concentrations of 0.01 and 0.1 *μ*M) significantly increased CYP26A1 expression compared to retinoid alone after 24 h (two-way ANOVA, effect of R116010 F_1,32_=15.496, *P*<0.001). Compared to either retinoid alone, R116010 enhanced CYP26A1 expression 6.8- and 1.7-fold with 0.01 and 0.1 *μ*M ATRA and 2.6- and 3.4-fold with 0.01 and 0.1 *μ*M 13cisRA (Figure 5D).

## DISCUSSION

Resistance to RA treatment may be related to drug metabolism and represents a significant clinical problem. Non-specific *P*450 inhibitors such as ketoconazole and liarozole can inhibit the formation of metabolites of RA and can exert ATRA-mimetic effects *in vivo* by enhancing endogenous plasma levels of ATRA (Van Wauwe *et al*, 1988, 1992). However, one of the limitations to the use of liarozole is its lack of specificity, and more selective inhibitors of RA metabolism are needed (reviewed in Njar, 2002). The current study was designed to determine whether agents that can inhibit the metabolism of ATRA in NBL cells may be able to increase the cellular response to RA.

It was hypothesised that high concentrations of acitretin may act to reduce ATRA metabolism by binding to CRABP I, therefore facilitating a greater availability of ATRA for binding to nuclear receptors. Incubation of SH-SY5Y cells with acitretin resulted in a concentration-dependent selective increase in intracellular ATRA levels, after incubation with either ATRA or 13cisRA. Further investigation in a panel of NBL cell lines revealed a mixed response to preincubation with acitretin. There was a selective increase in ATRA concentrations, but the degree of induction varied. Evaluation of CRABP protein expression in these cell lines demonstrated that CRABPs were expressed and could be induced by RA in SH-SY5Y cells. There was significant expression of the CRABPs in untreated NGP cells and this was further induced upon incubation with RA. However, there was a relatively low level of expression of CRABP in IMR-32 cells and none detected in SH-EP cells. Correspondingly, ATRA concentrations in SH-SY5Y cells increased significantly after pre-incubation with acitretin (50 *μ*M) followed by either ATRA or 13cisRA. This effect was also observed in IMR-32 and NGP cells, though the increases in intracellular ATRA concentrations were not significant. This general trend suggests that acitretin has a greater effect in cell lines that induce expression of CRABPs, supporting the hypothesis that the acitretin effects are mediated by CRABP I. However, the strong expression of the CRABPs in the NGP cell line indicates that the contribution of CRABP I in facilitating RA metabolism may vary between cell lines.

Acitretin itself induced the expression of CRABPs, CYP26A1 mRNA and an RARE-reporter construct, and had significant anti-proliferative effects in SH-SY5Y cells. Although acitretin binds poorly to the RARs that mediate transcription, it has previously been reported to activate RARs and to exert RA-mimetic effects (Tian *et al*, 1997; Saurat, 1999).

It has been suggested that CRABP I over-expression may increase initial intracellular RA concentrations (Chen *et al*, 2003). Furthermore, over-expression of CRABP I can enhance the RA induction of a reporter construct and cyclin D1 expression (Wei *et al*, 1999; Blaese *et al*, 2003), while antisense oligonucleotides to CRABP I partially inhibited the RA-induced expression of TGF*β*, RAR*β* and tenascin (Nugent and Greene, 1995). These observations indicate that CRABP I may exert bi-phasic effects on both intracellular RA concentrations and gene expression and suggest a role for this protein in the facilitation of signal transduction prior to any effect on RA degradation.

In SH-SY5Y cells transfected with RARE-SEAP, coincubation of acitretin and either ATRA or 13cisRA resulted in a lower degree of induction of SEAP expression compared to ATRA or 13cisRA alone (Figure 4), which may be a consequence of acitretin binding to either CRABP I or II. Supportive data were also obtained from CRABP II analysis, where the induction of protein expression was lower when comparing ATRA alone to ATRA in combination with acitretin. Recent data indicate a role for CRABP II in the formation of the RA–RAR complex, subsequently acting as a coactivator of RAR-mediated transcription (Dong *et al*, 1999; Budhu and Noy, 2002). Acitretin binding to CRABP II may, in part, be responsible for the reduction of RA-responsive gene expression by preventing CRABP II binding to the transcription complex. The ability of acitretin to increase intracellular concentrations of ATRA appears to override its capacity to stimulate the expression of proteins that degrade RA. In spite of this, it has proved difficult to analyse a biological downstream effect of increasing ATRA concentrations, due to the RA-mimetic effects of acitretin itself. Based on these data, it is possible that acitretin may provide a clinical benefit in NBL. However, due to the complex nature of this interaction and the more selective effects observed with R116010, further studies to determine the mechanism of the interaction between acitretin and RA would be necessary.

R116010 is an effective inhibitor of RA metabolism, with a 100-fold greater potency compared to that of liarozole in intact human breast T47D carcinoma cells. R116010 also has improved specificity, suggesting it is less likely to produce adverse side-effects. *In vivo*, R116010 itself inhibits the growth of murine oestrogen-independent TA3-Ha mammary carcinoma tumours at doses as low as 0.16 mg kg^−1^ and RA-like side effects are observed at higher doses (5 mg kg^−1^) (Van heusden *et al*, 2002).

At concentrations of both 1 and 10 *μ*M, R116010 induced significant increases in intracellular ATRA levels after incubation of SH-SY5Y cells with either ATRA or 13cisRA, consistent with an inhibition of ATRA metabolism. This increase was reflected in enhanced RA-induced gene expression and inhibition of cell proliferation when these cells were coincubated with R116010. Induction of the RARE-SEAP reporter gene increased 2.3- and 1.8-fold with ATRA and 13cisRA, respectively, in combination with 1 *μ*M R116010 compared to either agent alone, corresponding to increasing intracellular ATRA concentrations. Additionally, RA rapidly induced CYP26A1 expression in these cells, and this response was significantly increased by coincubation with R116010. Taken together, these data indicate that R116010 can inhibit ATRA metabolism, which may lead to an enhanced induction of retinoid responsive genes in human NBL cells *in vitro*. However, these data do not exclude alternative mechanisms of action for R116010.

It has previously been demonstrated that liarozole in combination with ATRA can partially reverse the decline of plasma ATRA concentrations in patients, without increased toxicity (Miller *et al*, 1994). A similar elevation in plasma ATRA concentrations was achieved with an intravenous (i.v.) administration of a liposomal formulation of ATRA, implying that reducing ATRA metabolism may be advantageous and potentially more effective for long-term treatment (Estey *et al*, 1996; Ozpolat *et al*, 2003). The high degree of interpatient variability in plasma 13cisRA concentrations suggests that inhibitors of RA metabolism may be beneficial to those patients with lower drug exposures. While changes to 13cisRA dose may be one solution to this problem, the use of agents such as R116010 may be more appropriate if lower clinical exposures are a result of higher rates of RA metabolism. Although two-fold increases in intracellular ATRA levels may not markedly increase the antiproliferative effect of RA in NBL, there may be a larger impact on other factors, such as the facilitation by RA of tumour lysis by cytotoxic lymphocytes (Vertuani *et al*, 2003).

The relationship between RA sensitivity and RA turnover appears to be complex, particularly following the identification of the RA-inducible *P*450 enzymes, and it is likely that these events are independently related to the expression of the RARs or other intracellular molecules that mediate RA response. It is possible that RA catabolism is an early event in the emergence of resistance to retinoids, and may precede the selection of tumour cells with modifications, such as MYCN amplification, that lead to enhanced survival. Retinoic acid resistance has been extensively studied in acute promyelocytic leukaemia, and many of these observations may translate to NBL. The observations reported here suggest a close link between induction of CYP26 and RA-mediated responses. However, the augmentation of these responses by R116010 is encouraging and points the way to further optimization of retinoid treatment in preclinical and clinical studies.

## Figures and Tables

**Table 1:** Intracellular retinoic acid concentrations following incubation with or without 50 *μ*M acitretin for 24 h prior to the addition of 10 *μ*M ATRA or 13cisRA for a further 24 h^a^

		**ATRA incubation (*μ*M)**			**13cisRA incubation (*μ*M)**		
**Cell line**	**RA isomer**	**Control**	**Acitretin**	***P* b**	**Control**	**Acitretin**	***P* b**
SH-SY5Y	ATRA	39.1±7.4	113.5±28.7	0.003	4.2±1.1	19.3±4.1	0.005
	13cisRA	7.6±1.3	7.5±0.7	0.744	25.4±4.1	28.7±6.9	0.674
							
SH-EP	ATRA	908.1±378	1050±439.3	0.652	49.9±10.6	51.9±18.2	0.933
	13cisRA	43.5±9.1	39.9±11.3	0.886	92.7±19.7	75.7±29.1	0.435
							
IMR-32	ATRA	354.6±31.6	447.9±83.6	0.382	59.9±31.1	120.2±66.2	0.707
	13cisRA	29.7±2.5	26.5±1.6	0.352	39.4±17.3	60.4±14.6	0.378
							
NGP	ATRA	613.3±165.3	1657±688.3	0.083	32.5±15.2	21.4±1.4	0.642
	13cisRA	46.0±11.7	67.2±23.4	0.41	84.6±11.4	106.9±7.7	0.221

a Results are expressed as mean±s.e.m. from *n*⩾3 experiments.

b Probabilities from two-sided *t*-tests.

**Table 2:** Measurement of the antiproliferative effect of ATRA and 13cisRA in the absence or presence of R116010 or acitretin in SH-SY5Y cells^a^

**Incubation**		
**RA isomer**	**Inhibitor (1 *μ*M)**	**Growth inhibition (% untreated cell growth)**
	R116010	96.4±2.4
	Acitretin	66.5±1.9
		
0.01 *μ*M ATRA	—	60.3±2.9
	R116010	48.9±2.2
	Acitretin	52.2±2.3
		
0.1 *μ*M 13cisRA	—	53.8±2.5
	R116010	42±1.9
	Acitretin	55.7±3.1

a Results are expressed as mean±s.e.m. from *n*⩾3 experiments.
